# A Successful Infliximab Treatment of a Pediatric Case of Severe Polyarteritis Nodosa With a Cerebral Infarction and a Decreased Adenosine Deaminase 2 Activity

**DOI:** 10.7759/cureus.47952

**Published:** 2023-10-30

**Authors:** Hiroki Izumo, Nobutsune Ishikawa, Yoshiyuki Kobayashi, Takehiko Doi, Satoshi Okada

**Affiliations:** 1 Department of Pediatrics, Hiroshima University Hospital, Hiroshima, JPN; 2 Department of Pediatrics, Hiroshima Prefectural Hospital, Hiroshima, JPN

**Keywords:** adenosine deaminase 2, tumor necrosis factor-α(tnf-α) inhibitor, childhood cerebral infarction, deficiency of adenosine deaminase 2, polyarteritis nodosa

## Abstract

Polyarteritis nodosa (PAN) is a systemic necrotizing vasculitis common in males over 50 years of age that causes various organ symptoms. In recent years, it has become important to distinguish deficiency of adenosine deaminase 2 (DADA2) from childhood-onset PAN.

A 13-year-old girl was urgently transferred to our hospital with sudden weakness in her right upper and lower limbs. The National Institutes of Health Stroke Scale (NIHSS) was 8. Plain MRI of the brain indicated high-signal areas in the right caudate nucleus, internal capsule, and left basal ganglia when applying T2-weighted, fluid-attenuated inversion recovery (FLAIR), and diffusion-weighted imaging (DWI); and low signals in the same regions in an apparent diffusion coefficient (ADC) map. It demonstrated inflammatory demyelinating disease of the central nervous system or multiple cerebral infarctions attributable to vasculitis, and it is difficult to differentiate between them based on image findings alone, and cannot be determined without following the clinical course. Hence, we treated with steroid therapy, which is effective for both conditions. Although the paralysis was alleviated, an MRI of the brain reperformed on day 7 revealed expansion of the lesion with contrast enhancement in the feeding area of the left lateral striatal artery, a high signal in DWI, and a low signal in an ADC map. Based on the clinical and radiological findings, we diagnosed a cerebral infarction attributable to vasculitis. Contrast computed tomography (CT) of her chest and abdominal CT angiography revealed that she met the diagnostic criteria for PAN, and adenosine deaminase 2 (AD2) activity level was low. The patient was treated with steroids combined with azathioprine and cyclophosphamide but three weeks after discharge developed a new cerebral infarction in the right basal ganglia. We commenced infliximab; no recurrence of cerebral infarction has been noted.

The low AD2 activity may explain the intractable atypical course of this case. Further studies are needed to reveal the role of AD2 in patients with residual enzyme activity and reevaluation of the PAN diagnostic criteria is essential.

## Introduction

Polyarteritis nodosa (PAN) is a systemic necrotizing vasculitis associated with lesions in both small and medium blood vessels. It is common in males over 50 years of age and can cause various organ symptoms. Steroids are the basic treatment [1,2]. In recent years, it has become important to distinguish PANs that develop in childhood from a deficiency of adenosine deaminase 2 (DADA2) because the treatments differ. DADA2 presents as PAN-like vasculitis symptoms but steroids are generally insufficient and tumor necrosis factor-α (TNF-α) inhibitors are required. Herein, we describe an atypical PAN case who presented with recurrent cerebral infarctions within a short time interval despite treatment with steroids combined with azathioprine and cyclophosphamide. She fulfilled the diagnostic criteria for PAN and concomitantly exhibited low-level adenosine deaminase 2 (AD2) activity; she required infliximab to control her disease. Her clinical course was not typical of pediatric PAN as she had had cerebral infarctions in childhood and platelet levels did not increase [2,3]. Our case raises a critical issue in terms of the diagnosis of childhood PAN with low-level AD2 activity. We describe the clinical course of the severe neurological symptoms and discuss the spectrum of disease and the importance of accurate diagnosis.

## Case presentation

A 13-year-old girl suddenly developed weakness in the right upper and lower limbs on the day before she was transferred to our hospital. She had noticed livedo on her thigh four years prior but had not undergone a medical evaluation. Her father developed an acute subarachnoid hemorrhage in his 40s without sequelae but no other symptom suggestive of vasculitis was found. Magnetic resonance imaging (MRI) of her brain in the first hospital revealed high-intensity areas in fluid-attenuated inversion recovery (FLAIR) images of the right caudate nucleus to the internal capsule and left basal ganglia.

**Figure 1:**
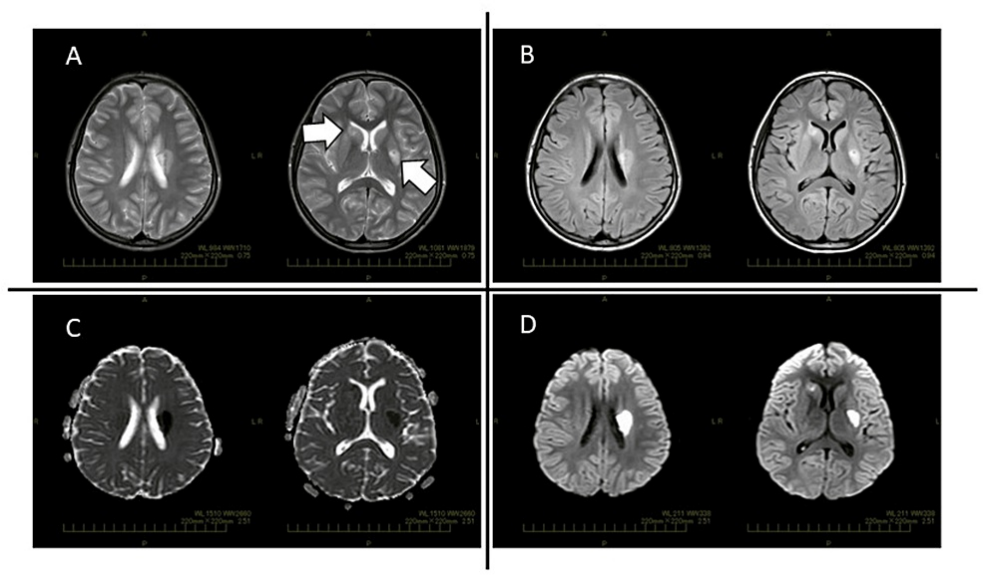
Plain MRI of the patient's brain in our hospital Plain MRI of the brain indicated high-signal areas in the right caudate nucleus, internal capsule and left basal ganglia when applying T2-weighted, fluid-attenuated inversion recovery (FLAIR), and diffusion-weighted imaging (DWI); and low signals in the same regions in an apparent diffusion coefficient (ADC) map. (A) T2-weighted images, (B) FLAIR, (C) DWI, (D) ADC map. White arrows point to the lesions.

Upon admission to our hospital, her blood pressure was 144/97 mm Hg, pulse 84/min, respiratory rate of 20/min with an O2 saturation of 98% on room air and temperature of 36.8°C. She exhibited weakness in the right upper and lower limbs, but she was of normal consciousness and able to speak and walk with a limp on the right leg. The National Institutes of Health Stroke Scale (NIHSS) was 8 and the modified Rankin Scale (mRS) was 4. The laboratory values were found to be white blood cell count of 9.1 × 103/µL (normal range: 3.3-8.6 × 103/µL), haemoglobin of 12.6 g/dL (normal range: 11.6-14.8 g/dL), platelet count of 339 × 103/µL (normal range: 158-348 × 103/µL), C-reactive protein (CRP) of 5.59 mg/dL (normal range: 0-0.14 mg/dL), serum amyloid A (SAA) of 451 μg/mL (normal range: 0-8 μg/mL) and 50% hemolytic complement activity (CH50) of 58.4 CH50/mL (normal range: 30-46 CH50/mL). Liver and renal function were normal. Antinuclear antibody, antineutrophil cytoplasmic antibody, anti-aquaporin 4 antibody and anti-myelin-oligodendrocyte glycoprotein antibody tests were negative. No hepatitis B or C infection was noted. Although human immunodeficiency virus status was not examined, she exhibited no symptoms of infection. The laboratory values of cerebrospinal fluid were found to be cell count of 1/µL (normal range: 0-5/µL), total protein of 23 mg/dL (normal range: 15-45 mg/dL), glucose of 48 mg/dL (normal range: 50-80 mg/dL), immunoglobulin G index of 0.49 (normal range: less than 0.8), and myelin basic protein and oligoclonal band were negative. Urine analyses were normal. Plain MRI of the brain indicated high-signal areas in the right caudate nucleus, internal capsule, and left basal ganglia when applying T2-weighted, FLAIR, and diffusion-weighted imaging (DWI); and low signals in the same regions in an apparent diffusion coefficient (ADC) map (Figure 1). Nerve conduction studies were not performed.

We considered central nervous system inflammatory demyelinating disease and cerebral infarction. We prescribed oral antiplatelet drugs and steroid pulse therapy. Although the clinical symptoms improved rapidly, an MRI of her brain on day 7 of hospitalization showed expansion of the lesion, which exhibited high signals in DWI and low signals in an ADC map. Gadolinium angiography confirmed the contrast enhancement findings in the left lateral striatal artery. Based on the clinical and radiological findings, we diagnosed cerebral infarction attributable to vasculitis. Both splenic and renal infarctions were observed in contrast-enhanced CT of the torso performed on the same day. On day 17 of hospitalization, abdominal CT angiography revealed an aneurysm in the peripheral small artery of the right kidney. These findings supported the diagnosis of systemic vasculitis. At this time, she met the diagnostic criteria for childhood PAN established by the European League Against Rheumatism/Paediatric Rheumatology International Trials Organisation/Paediatric Rheumatology European Society, and “probable” PAN as defined by the Ministry of Health, Labor, and Welfare of Japan [4,5] (Table 1); the AD2 activity was about half that of healthy controls, equivalent to that of a heterocarrier. Therefore, a diagnosis of DADA2 was not made at this time. After she was diagnosed with severe PAN, treatment with an oral steroid (prednisolone: 0.5mg/kg/day) combined with monthly intravenous administration of cyclophosphamide (CPA: 500mg/m^2^) was commenced [1]. She was discharged from our hospital on day 51 after the clinical and blood test findings improved. At discharge, she was able to perform most activities on her own. NIHSS was 0 and mRS was 1. However, after discharge administration of azathioprine (AZP: 0.6mg/kg/day) was started as an increase in SAA levels was observed, considering the effect on blood pressure, the dose of prednisolone was not increased. On day 71 after the initial hospitalization, she developed a new cerebral infarction in the right basal ganglia. Infliximab was commenced. She gradually improved, although her limb muscle strength remained weak and dysphonia remained at the time of the second discharge, after which regular infliximab was continued. No recurrence of the cerebral infarction or any side effects has been observed up until this writing (currently 12 months after infliximab commencement).

**Table 1:** Diagnostic criteria for childhood PAN established by EULAR/PRINTO/PRES and Ministry of Health, Labor and Welfare of Japan. Italics indicate findings consistent with our case. c-PAN: childhood polyarteritis nodosa, EULAR: European League Against Rheumatism, PRINTO: Paediatric Rheumatology International Trials Organisation, PRES: Paediatric Rheumatology European Society, GFR: glomerular filtration rate

・Diagnostic criteria for childhood PAN established by EULAR/PRINTO/PRES	・Diagnostic criteria for PAN established by the Ministry of Health, Labor and Welfare of Japan										
(1) Criteria	(1) Major symptoms										
1. Skin involvement	1. Fever (38°C or higher, two weeks or longer) and weight loss (6 kg or higher within six months)										
2. Myalgia or muscle tenderness	2. Hypertension										
3. Hypertension	3. Rapidly progressing renal failure and renal infarction										
4. Peripheral neuropathy	4. Cerebral hemorrhage, cerebral infarction										
5. Renal involvement	5. Myocardial infarction, ischemic heart disease, pericarditis, heart failure										
(2) Histopathology	6. Pleuritis										
Evidence of necrotising vasculitis in medium-sized arteries.	7. Gastrointestinal bleeding, intestinal obstruction										
(3) Angiographic abnormalities	8. Mononeuritis multiplex										
Angiography showing aneurysm, stenoses or occlusion of a medium or small-sized artery, not due to fibromuscular dysplasia, or other non-inflammatory causes.	9. Subcutaneous nodule, skin ulcer, gangrene, purpura										
	10. Polyarthralgia (polyarthritis), muscle pain (myositis), muscle weakness										
Definite: Histopathology or* angiographic abnormalities (mandatory) plus one of the five criteria.*	(2) Histological findings										
	Presence of fibrinoid necrotizing vasculitis of medium and small arteries										
	(3) Angiographic findings										
	*Multiple small aneurysms and stenosis/occlusion of abdominal aortic branches (especially intrarenal arterioles)*										
	Definite: Cases with two or more major symptoms and histological findings. Probable*: (a) Cases with two or more major symptoms and angiographic findings *or (b) Cases with six or more major symptoms including fever.										

**Figure 2:**
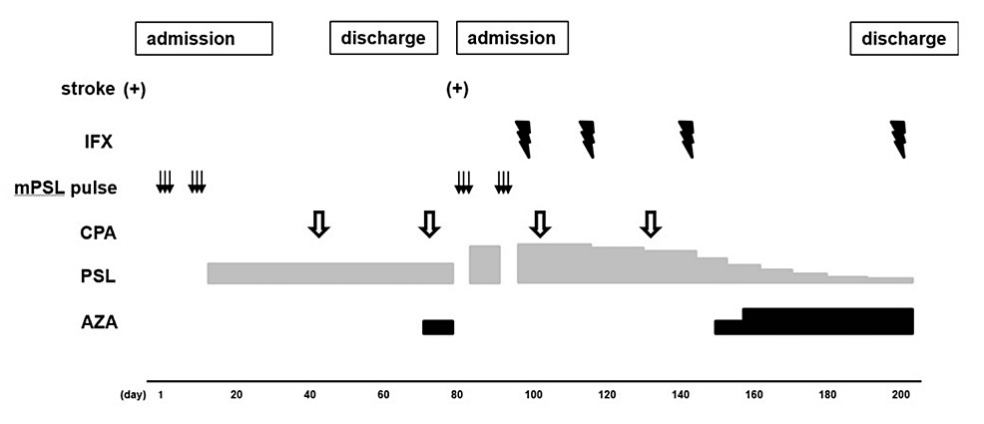
The clinical course mPSL: methylprednisolone, CPA: cyclophosphamide, IFX: infliximab, PSL: prednisolone, AZA: azathioprine

Figure 2 shows the clinical course. Genetic testing identified missense mutations c.1358A>G (p.Tyr453Cys) and c.1065C>A (p.Phe355Leu) in the AD2 gene, which are tagged as Pathogenic and of Uncertain_significance in ClinVar, respectively. The patient’s parents did not consent to genetic analysis for themselves except the patient. We obtained the ethical committee approval and informed consent of the patient's parents for off-label using of infliximab. We obtained the informed consent of the patient’s parents for this publication.

## Discussion

The most common causes of cerebral infarction in children are cardioembolic, moyamoya disease, craniocervical artery dissection, and congenital coagulation disorders; autoimmune vasculitis inflammatory diseases are relatively rare [6]. The treatment strategy for an ischemic stroke attributable to autoimmune vasculitis inflammatory disease differs radically from treatments for other causes of vascular obstruction. Thus, it is very important to consider the possibility of autoimmune vasculitis inflammatory disease when a child has a stroke. In our present case, the multiple cerebral lesions with a background of elevated inflammatory markers and abnormal vessel findings on gadolinium-enhanced head MRI suggested that an autoimmune vasculitis inflammatory disease might have caused the cerebral infarction. We diagnosed PAN after systemic evaluation.

PAN is a rare disease that affects small and medium blood vessels, and causes systemic necrotizing vasculitis. Typical cases present with systemic symptoms including fever, weight loss, myalgia, and arthralgia. It can cause multiple lesions in various internal organs including the heart, kidneys, central/peripheral nerves, respiratory system, and gastrointestinal tract. Sometimes skin symptoms are observed. Cyclophosphamide and glucocorticoids should be given to PAN cases with severe conditions that threaten organs or life [1,7]. In addition, several reports have indicated that infliximab or tocilizumab is required to treat steroid-resistant PAN [8-13].

In recent years, the importance of differentiating childhood-onset PAN from DADA2 has become clearer. DADA2 is an autosomal-recessive disease caused by AD2 loss-of-function and cases present with vasculitis symptoms similar to those of PAN (infarction, fever, skin reticularis, and skin ulcers). Some cases develop hypoglobulinemia and cytopenia. Diagnosis is based on clinical symptoms, measurement of AD2 activity, and genetic testing. As treatments, TNF-α inhibitors are preferred to prevent stroke, although no consensus treatment strategy exists. Our present case met the PAN diagnostic criteria after a detailed examination of the vascular imaging findings. At that time, she did not fulfil the current criteria for DADA2 because the AD2 activity level was that of a heterocarrier. Cerebral infarctions develop in 2-10% of all PAN cases but childhood stroke is rather characteristic of DADA2 [2,3]. Kasap et al. reported that a PAN group featured significantly fewer cases of cerebral infarction and lymphopenia than a DADA2 group but a significantly higher number of cases with increased platelet levels during the active phase [2]. No thrombocytosis was observed during the clinical course of our patient. In one study, Erden et al. reported that juvenile-onset PAN was associated with significantly fewer renal and neurological symptoms than adult-onset PAN, suggesting that juvenile PAN might have a more benign course [14]. Thus, our case showed atypical childhood-onset PAN in terms of the clinical course, including high resistance to conventional treatment. Of note, our case also exhibited the characteristics of DADA2 and was successfully treated with the TNF-α inhibitor infliximab.

A report that a sibling with the same missense mutation c.1358A>G (p.Tyr453Cys) developed cerebral infarction in adulthood suggests that decreased AD2 activity can affect the disease course [15]. Currently, the disease risk in DADA2 carriers is unclear; the gene-dose effect decreases enzyme activity. Such reduced activity in combination with other predisposing genetic and environmental factors may decrease the threshold for inflammatory manifestations [16]. Although genetic testing of her father was not conducted, his history of a subarachnoid haemorrhage at a relatively young age may suggest an influence of genetic factors on her condition. Lee et al. examined cases with definitive diagnoses of DADA2 and reported that gene mutations associated with some residual in vitro enzymatic activity also caused vasculitis [17]. The current diagnostic criteria for and treatment of severe childhood PAN should be reevaluated to include an analysis of the AD2 activity level.

## Conclusions

As pediatric patients with severe PAN symptoms such as early cerebral infarctions are at high risk for a poor prognosis and lower quality of life, it is important to consider the possible role of decreased AD2 activity, and promptly measure that activity. Further studies are needed to reveal the role of AD2 in patients with residual enzyme activity, and reevaluation of the PAN diagnostic criteria is essential.
